# Dynamic Meso-Scale Anchorage of GPI-Anchored Receptors in the Plasma Membrane: Prion Protein vs. Thy1

**DOI:** 10.1007/s12013-017-0808-3

**Published:** 2017-06-24

**Authors:** Yuri L. Nemoto, Roger J. Morris, Hiroko Hijikata, Taka A. Tsunoyama, Akihiro C. E. Shibata, Rinshi S. Kasai, Akihiro Kusumi, Takahiro K. Fujiwara

**Affiliations:** 1 Center for Meso-Bio Single-Molecule Imaging (CeMI), Institute for Integrated Cell-Material Sciences (WPI-iCeMS), Kyoto University, Kyoto, 606-8507 Japan; 2 Institute for Frontier Life and Medical Sciences, Kyoto University, Kyoto, 606-8507 Japan; 3 Membrane Cooperativity Unit, Okinawa Institute of Science and Technology, Okinawa, 904-0495 Japan; 4 Department of Chemistry, King’s College London, London, SE1 1DB UK

**Keywords:** GPI-anchored receptor, Prion protein, Thy1, Cluster, Diffusion, Single fluorescent-molecule imaging and tracking

## Abstract

The central mechanism for the transmission of the prion protein misfolding is the structural conversion of the normal cellular prion protein to the pathogenic misfolded prion protein, by the interaction with misfolded prion protein. This process might be enhanced due to the homo-dimerization/oligomerization of normal prion protein. However, the behaviors of normal prion protein in the plasma membrane have remained largely unknown. Here, using single fluorescent-molecule imaging, we found that both prion protein and Thy1, a control glycosylphosphatidylinositol-anchored protein, exhibited very similar intermittent transient immobilizations lasting for a few seconds within an area of 24.2 and 3.5 nm in diameter in CHO-K1 and hippocampal neurons cultured for 1- and 2-weeks, respectively. Prion protein molecules were immobile during 72% of the time, approximately 1.4× more than Thy1, due to prion protein’s higher immobilization frequency. When mobile, prion protein diffused 1.7× slower than Thy1. Prion protein’s slower diffusion might be caused by its transient interaction with other prion protein molecules, whereas its brief immobilization might be due to temporary association with prion protein clusters. Prion protein molecules might be newly recruited to prion protein clusters all the time, and simultaneously, prion protein molecules in the cluster might be departing continuously. Such dynamic interactions of normal prion protein molecules would strongly enhance the spreading of misfolded prion protein.

## Introduction

In recent years, various membrane proteins and lipids in the plasma membrane (PM) have been found to undergo temporary entrapment or immobilization in meso-scale (a few nm ~ a few 100 nm) domains [1–8]. Importantly, the occurrences of such transient anchorages are not limited to raft-associated molecules. Non-raft molecules often exhibited temporary immobilization/entrapment in meso-scale domains and molecular clusters in the PM [3, 5, 9].

Sahl et al. [3] found that fluorescently-labeled sphingomyelin and phosphatidylethanolamine (with a hydrophobic ATTO647N dye in the headgroup), which both turned out to behave like non-raft lipids according to Sezgin et al. [10] including the same authors, exhibited alternating periods of rapid diffusion and transient cholesterol-dependent entrapment/anchorage in areas of ~6 nm in diameter, with average trapping times of ~15 and <4 ms, respectively, at ~23 °C in the PM of living PtK2 cells (70 and 30% entrapments over all of the trajectory lengths, respectively). In contrast, Honigmann et al. [5], using the same ATTO647-sphingomyelin (a non-raft lipid probe), found that the immobilization of the sphingomyelin analog in domains with sizes smaller than 80 nm in diameter, and in the time scale of ~10 ms in the PM of PtK2 cells, was not due to the presence of raft-based nanodomains, but to alternative interactions that are perhaps based on the sphingosine backbone structure.

Transient entrapment/anchorage might be critically linked to the biological functions of membrane receptors and other signaling molecules and to the formation of signal transduction platforms in the PM [1, 2, 11–13]. Suzuki et al. [1, 2] reported that upon the engagement or colloidal gold-induced cross-linking of physiological levels of CD59, a glycosylphosphatidylinositol-anchored receptor (GPI-AR), CD59 clusters containing an average of about five CD59 molecules underwent alternating periods of slow diffusion (1.2-s lifetime) and actin-dependent temporary immobilization called “Stimulation-induced Temporary Arrest of LateraL diffusion” (STALL) (0.57-s lifetime) in areas of ~50 nm in diameter (20–40% of the entire trajectory length). Each CD59 cluster continually recruits molecules of the Src-family kinase Lyn, with a residency time of ~0.2 s. Gαi2, a subunit of a trimeric G protein, was also transiently recruited to CD59 clusters (~0.13-s lifetime), triggering the binding of the CD59 cluster to a cortical actin filament, i.e., the STALLing of the CD59 cluster (perhaps due to the Gαi2-mediated activation of Lyn, which is almost always located in the cluster). The recruitment of Gαi2 and Lyn depended on both protein–protein and raft–lipid interactions. Furthermore, PLCγ2 molecules were transiently (~0.25 s) recruited from the cytoplasm to CD59 clusters exclusively during its STALLing periods, to produce IP_3_, followed by Ca^2+^ mobilization. These results suggest that CD59 clusters undergoing STALL may be a key, albeit transient, platform for transducing the extracellular GPI-AR engagement signal to the intracellular IP_3_–Ca^2+^ signal, via PLCγ2 recruitment.

Transient entrapment in meso-scale protein clusters and domains might also be critically linked to pathological processes, such as the development of prion diseases. Mammalian prion diseases involve the aggregation of misfolded prion protein (PrP), a GPI-AR. The central mechanism for the transmission of the PrP misfolding is the structural conversion of the normal cellular PrP to the pathogenic misfolded PrP, by the interaction with misfolded PrP [14]. Furthermore, it was proposed that the aggregation of misfolded PrP may proceed from many different precursors, which might include normal PrP [15]. Therefore, if normal PrP molecules tend to be temporarily immobilized at PrP clusters, it would become an important mechanism for the very fast spreading of misfolded PrP [16].

Indeed, all of the GPI-ARs examined previously, including Thy1, CD59, DAF, and GFP-GPI, were found to frequently form transient homodimers, which work as units for raft organization and function, with lifetimes on the order of 0.2 s [11]. Furthermore, PrP and Thy1 reportedly formed larger clusters, but quite independently [17, 18]. The immunoelectron microscopy assay with monovalent immunogold labeling revealed that the clusters of the two proteins mostly existed in separate domains on the neuronal cell surface: 86% of PrP was clustered in domains lacking Thy1, although 40% of Thy1 had a few molecules of PrP associated with it. Only 1% of all clusters contained appreciable levels of both proteins (>3 immunogold labels for both). If these two molecules form clusters primarily by raft–lipid interactions, then such separate clustering would not be possible [19–21]. Therefore, the immunoelectron data suggest that even normal PrP tends to form homo-oligomers due to interactions of PrP’s protein domain (the raft–lipid interaction is less involved, because Thy1–Thy1 and Thy1–PrP clustering occur infrequently).

Meanwhile, our knowledge about how GPI-ARs undergo temporary immobilization/anchorage in the PM before ligation-activation is quite limited. In HeLa cells, ~20% (time fraction against the entire length of observed trajectories) of a GPI-AR, Thy1, was found to undergo temporary immobilization in the PM, which was about the same time fraction of temporary immobilization observed for transferrin receptor molecules transiently trapped in clathrin-coated pits [22].

Therefore, the goals for the present research were to reveal whether the non-engaged PrP and Thy1 undergo distinctively different temporary entrapment behaviors in the PM, and to determine whether the behaviors are different in neurons (because both PrP and Thy1 are endogenously enriched in neurons) and in cell lines derived from non-neuronal cells (CHO-K1 cells). Furthermore, neurons undergo dramatic changes during the first 2 to 3 weeks in culture, in terms of their structures and neuronal activities [23–26]. Therefore, in the present study, we comprehensively examined the dynamics of non-engaged PrP and Thy1 and their immobilization/anchorage/entrapment behaviors in the PM using single fluorescent-molecule tracking [22, 27, 28]. We compared their behaviors in the PM of primary cultured hippocampal neurons from new-born rats for 1 and 2 weeks in vitro (7–8 and 14 days in vitro [DIV]) with those in the PM of CHO-K1 cells.

## Materials and Methods

### Plasmid Construction

The cDNA encoding ACP-PrP was constructed by fusing the cDNA for the signal sequence of PrP (22 amino acids) to the cDNA encoding the ACP-tag protein (Covalys), which was subsequently fused to a 69 base linker (23 amino acids, with the sequence LDLIEGRGIPRNSRVDAGQASNS), and then to the cDNA encoding PrP (cloned from mouse brain). The cDNA encoding ACP-Thy1 was constructed by fusing the cDNA for the signal sequence of Thy1 (19 amino acids) to the cDNA encoding the ACP-tag protein (Covalys), which was subsequently fused to a 63 base linker (21 amino acids, with the sequence LDLIEGRGIPRNSRVDAGQAS), and then to the cDNA encoding Thy1 (cloned from rat brain).

### Cell Culture, Transfection, and Fluorescence Labeling

CHO-K1 cells were grown in Ham’s F12 medium (Sigma-Aldrich), supplemented with 10% fetal bovine serum (Sigma-Aldrich). The cells were transfected with the cDNA encoding ACP-PrP or ACP-Thy1 using Lipofectamine 2000 (Invitrogen), according to the manufacturer’s recommendations. Cells were plated on the glass bottoms (12-mm-∅ coverslips) of 35-mm-∅ glass-base dishes (Iwaki) and cultured under a 5% CO_2_ atmosphere at 37 °C.

Hippocampi from newborn Wister rats were isolated and dissociated within 24 h after birth, as described [24]. As a result, DIV approximately matches the age of a newborn rat. However, the developmental processes in vitro may be different from those in vivo. Neurons were cultured on the glass bottoms (12-mm-∅ coverslips) of 35-mm-∅ glass-base dishes (Iwaki). The cover glass was precoated with poly-L-lysine (Sigma-Aldrich). To facilitate the growth of neurons, rat Type-I astrocytes (~3.0 × 10^4^ cells/dish) were grown at the rim of the cover glass, to entirely surround it, for 1–2 days in Dulbecco's modified Eagle's medium (Sigma-Aldrich) containing 15% fetal bovine serum, before the initiation of the neuronal culture. Before plating the neurons, they were transfected with the cDNA encoding ACP-PrP or ACP-Thy1 by electroporation (Amaxa Nucleofector, Lonza), resuspended in minimum essential medium containing 5% fetal bovine serum, 2% B27 supplement (Sigma-GIBCO), and 0.5 mM glutamine, and then plated on the glass-bottom part of the culture dish at densities of 1.4–1.8 × 10^5^ cells/cm^2^ (due to electroporation, not all cells were alive). After culturing the cells under a 5% CO_2_ atmosphere at 37 °C for 24 h, DL-amino-5-phosphonovaleric acid (Sigma-Aldrich) was added at a final concentration of 5 µM. The neurons were further cultured for an additional 6–7 days or 13 days (7–8 or 14 DIV, respectively). All of the microscopic observations of neurons were conducted using these 7–8 or 14 DIV hippocampal neurons, and called 1- and 2-wk neurons, respectively, in this report.

ACP-PrP and ACP-Thy1 expressed in the PMs of CHO-K1 cells and hippocampal neurons were fluorescently labeled by incubating the cells with 50 nM ATTO594-CoA (custom synthesized from ATTO594-maleimide [ATTO-TEC GmbH] and CoA-SH [Covalys] by Shinsei Kagaku) and phosphopantetheine transferase (Covalys) at 37 °C for 5 min, followed by washing. Microscopy observations were performed in HBSS containing 2 mM PIPES (CHO-K1 cells) or in ACSF containing 10 mM HEPES (neurons), buffered at pH 7.4.

Due to the following very rough estimates of the expression levels, we believe that ACP-PrP and ACP-Thy1 in 1- or 2-wk neurons are not overexpressed. The mean number of Thy1 molecules per TRl4 neuroblastoma cell is approximately 2.25 × 10^5^ [29], whereas Thy1 is ~10-fold more abundant than PrP in the whole brain [18]. Assuming that 1- and 2-wk neurons express endogenous PrP and Thy1 at concentrations averaged over the whole brain, and that their expression levels of endogenous PrP and Thy1 are similar to those in TRI4 neuroblastoma cells, we estimate that the neuron we used expresses about 20,000 PrP and 200,000 Thy1 copies/cell. Meanwhile, under our observation conditions, 5–10% of ACP-PrP and ACP-Thy1 molecules were expected to be fluorescently labeled, and we observed under the microscope the cells that exhibited 0.19–0.27 single-molecule fluorescent spots/µm^2^. This suggests that the maximal expression levels of ACP-PrP and ACP-Thy1 in 1- and 2-wk neurons were 5.4 molecules/µm^2^ in the cell body (soma) region of the neuron. Although it is difficult to correctly evaluate the surface area in the cell body region, we assume that it is like a disk of 10 µm in diameter, which will have a surface area of ~160 µm^2^. We further assume that this represents 1/10 of the entire cell surface, providing an entire surface area of ~1600 µm^2^ (probably close to the maximal evaluation). These estimates give a copy number of ACP-PrP and ACP-Thy1 molecules in a single cell of ~9000 (probably close to the maximal estimate), which is very approximately about half the number of endogenous PrP and ~5% of the number of endogenous Thy1 in a neuron.

When the behaviors of PrP and Thy1 after homo-crosslinking them were observed in 1-wk neurons, we employed the following strategy. First, instead of the ACP-tag, we attached the Halo-tag to PrP and Thy1 at the same locations, and the expressed Halo-PrP and Halo-Thy1 molecules were labeled with tetramethylrhodamine (TMR). We observed if and how the dynamics of these molecules were modified by the addition of polyclonal anti Halo-tag antibodies (Promega, G928A), which would crosslink Halo-PrP and Halo-Thy1 molecules probably at similar levels (the expression levels of these two molecules were maintained quite similar to each other). In these experiments, switching of the tag protein from ACP to Halo was necessary due to the availability of good antibodies to Halo but not those to ACP.

### Single Fluorescent-Molecule Imaging

Molecules fluorescently labeled with ATTO594, located on the bottom PM (which faces the coverslip), were observed at 37 °C, using a home-built objective lens-type total internal reflection fluorescence microscope, based on an inverted microscope (Nikon, ECLIPSE Ti-E), as described previously [22, 30, 31]. The bottom PM was locally illuminated with an evanescent field (15 µm diameter; 100×, 1.49 NA objective lens, 400× total magnification). The fluorescence images of ATTO594 were projected onto the two-stage microchannel plate intensifier (C8600-03; Hamamatsu Photonics), coupled to a specially designed CMOS sensor-based camera (Photron, Tokyo, Japan) by way of an optical-fiber bundle, operated at a frame rate of 125 Hz (a time resolution of 8 ms). The recording duration was generally between 4 and 10 s (500–1250 frames).

### Single Molecule Tracking Analysis and “Temporary Arrest of LateraL diffusion (TALL)” Detection

For the analysis of single-molecule trajectories, all of (and only) the fluorescent spots that existed in the first recorded frame were tracked. The fluorescent spots that could be detected for at least 26 frames (at least for 208 ms or 25 steps in the trajectory) were used for further quantitative analysis [32, 33]. The trajectories were obtained using in-house software [34, 35].

The occurrences of TALLs were detected in single-molecule tracking trajectories, using the algorithm developed by Sahl et al. [3], with the parameter set (the most relevant parameter here was the threshold duration, which was set to 25 frames or for 200 ms) used previously [22]. The application of these parameters to the computer-generated simple-Brownian trajectories obtained by using the diffusion coefficients of 0.37 and 0.90 µm^2^/s, which were the minimal and maximal median diffusion coefficients found for molecules during the mobile periods in the present study, revealed that the false TALL events represented 0.03–6.9% of the total length of the Brownian trajectories.

## Results

### Both PrP and Thy1 Exhibited TALL, Repeated Transient Immobilization

All microscope observations were conducted at 37 °C, and all statistical examinations were performed using the Mann-Whitney *U*-test. Both PrP and Thy1 were conjugated with the ACP-tag protein at their N-termini (ACP-PrP and ACP-Thy1, respectively) at the cDNA level. Hetz et al. [36] showed that the PrP conjugated by GFP at PrP’s codon 25 was functional in terms of PrP trafficking in Neuro2A cells. We inserted the tag protein ACP between PrP’s codons 22 and 23. Therefore, we consider that the ACP-PrP molecules are functional, although it is not a proof. CHO-K1 cells and primary neurons cultured on coverslips of glass-base dishes were transfected with the appropriate cDNAs, and then ACP-PrP and ACP-Thy1 expressed on the cell surface were covalently labeled with the ATTO594-conjugated acyl-CoA ligand. Using a home-built TIRF microscope [37], we imaged ATTO594-labeled ACP-PrP and ACP-Thy1 at the level of single molecules, at an observation frame rate of 125 Hz (time resolution of 8 ms). In the present research, all of the observations were conducted for molecules existing in the basal PM of CHO-K1 cells and that of the cell body (soma) of primary–cultured hippocampal neurons obtained from new-born rats cultured for 7–8 DIV and 14 DIV (1-wk and 2-wk neurons, respectively). For concise presentation, ACP-PrP and ACP-Thy1 will simply be called PrP and Thy1, respectively, in the present report unless otherwise specified.

**Fig. 1:**
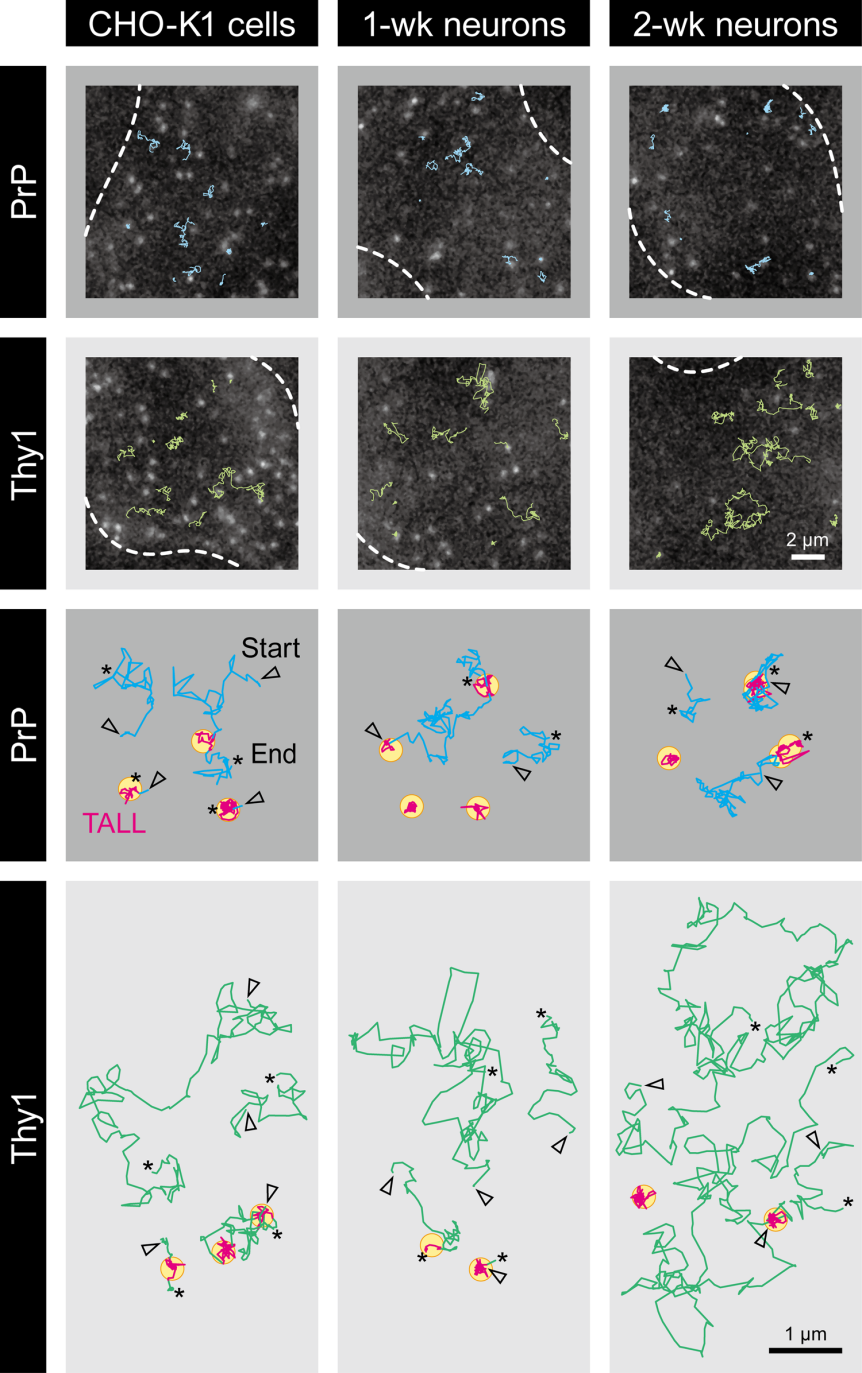
Typical snapshots of single fluorescent-molecule images of ACP-PrP and ACP-Thy1 labeled with ATTO594, and their trajectories in the PMs of CHO-K1 cells and 1- and 2-wk hippocampal neurons in culture. PrP undergoes frequent TALL events, more often than Thy1, and during the mobile periods, PrP diffusion is generally slower than Thy1 diffusion

Typical single-molecule images and trajectories of PrP and Thy1 are shown in Fig. 1. We believe that ACP-PrP and ACP-Thy1 in 1- or 2-wk neurons are not overexpressed: the expression levels of ACP-PrP might be about half that of endogenous PrP, whereas those of ACP-Thy1 might be ~5% of those of endogenous Thy1. See Materials and Methods (Cell Culture, Transfection, and Fluorescence Labeling). Single molecules of PrP and Thy1 were found to generally undergo thermal diffusion, but notably, they frequently became temporarily immobilized. Such events of temporary immobilization have previously been observed, and they were called “Temporary Arrest of LateraL diffusion (TALL)” (we use this term, because we previously introduced the term Stimulation-induced TALL or STALL). TALLs were detected by using the algorithm developed by Sahl et al. [3], with slight modifications (see Materials and Methods). TALLs were detected in the CHO-K1 cells and the hippocampal neurons.

Single-molecule trajectories were classified into three different categories: (1) the all-time mobile category, (2) the mobile + TALL category, representing trajectories including both the mobile and the TALL periods, and (3) the all-time immobile category. The trajectories that were classified into the all-time mobile category and the mobile parts of the trajectories that were classified into the mobile + TALL category were pooled, for analysis as the trajectories of mobile periods.

The TALL trajectories (the immobile part of category 2) and the all-time immobile trajectories (category 3) were also pooled as trajectories of immobile periods, for the following reason. Under the conditions where the entire observation period for each view field (500–1250 frames or 4–10 s in the present research) is substantially longer than the photobleaching lifetime of the employed fluorescent dye (138 frames or 1.10 ± 0.037 s for ATTO594 employed here), the distributions of the durations of TALL events and all-time immobile events will be the same, following the kinetics of *k*_M_ + *k*_photobleaching_ (where *k*_M_ represents the kinetic constant for the transition from the immobile state to the mobile state, and *k*_photobleaching_ represents the kinetic constant for photobleaching. As this kinetics can be written as the simple sum of *k*_M_ + *k*_photobleaching_, the distributions of the durations of TALL events and all-time immobile events become the same). This means that the all-time immobile trajectories found here were apparently-all-time-immobile molecules. Namely, they simply represent the molecules that were immobile (undergoing TALL) at time 0 and became photobleached before resuming diffusion. Therefore, the TALL trajectories (immobile parts of the trajectories in category 2) and the all-time immobile trajectories (category 3) were pooled as the trajectories of immobile periods in the present research (Some molecules that were immobile for much longer periods might exist, and would appear to be all-time immobile molecules in the present observation, without exhibiting TALL events. However, we did not find any such molecules that exhibited much longer all-time immobile durations).

Note that we did not detect any sign of hop diffusion at this observation rate [38, 39]. This is because the present observation frame rate (125 Hz) was too slow to detect hop diffusion. Previously, hop diffusion was observed at frame rates of 40,000 and 50,000 Hz, which are faster than the present rates by factors of 320–400 [33, 34, 40].

### PrP Exhibited Larger Time Fractions of Immobile Periods than Thy1

**Fig. 2:**
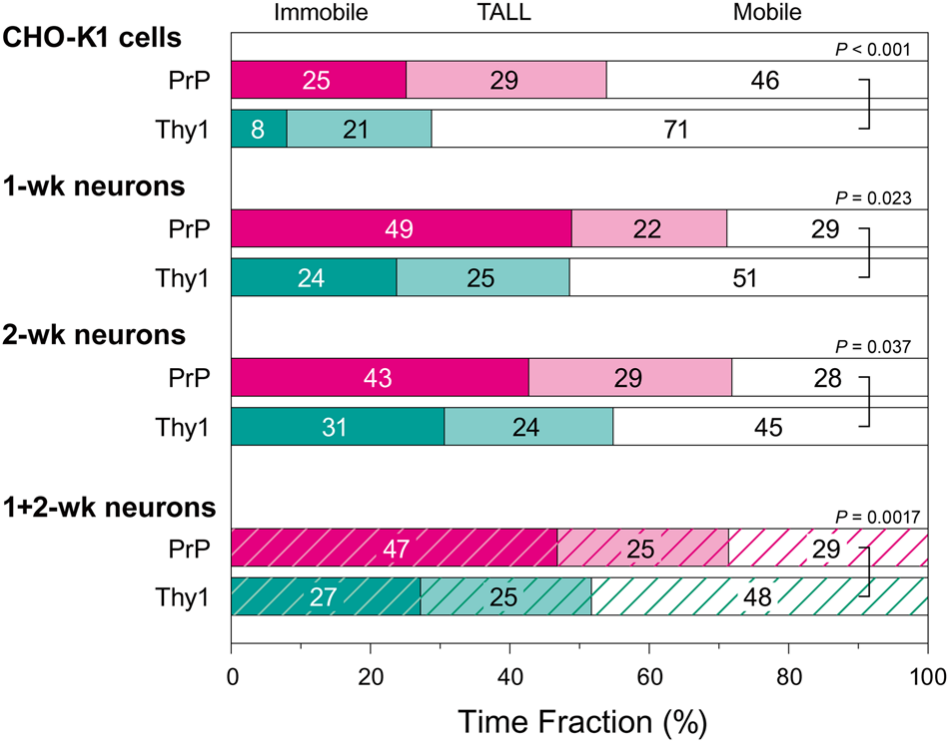
The time fractions of the mobile and immobile (all-time immobile + TALL) periods for PrP and Thy1. The time fractions of the mobile periods (and thus the immobile periods) for 1- and 2-wk neurons were not statistically significantly different (for both PrP and Thy1), and therefore, the data for 1- and 2-wk neurons were combined in the *bar graph* shown at the *bottom*. The large time fraction (72%) of the PrP immobile periods in 1 + 2-wk neurons is notable. In all cells, PrP exhibited fewer mobile periods than Thy1. Further details of the time fraction data are shown in Table 1

The time fractions of the mobile and immobile periods of PrP and Thy1 are shown in Fig. 2. Surprisingly, even in CHO-K1 cells, PrP was immobile (all-time immobile + TALL periods; in the following part, we use the term “immobile” in the sense of all-time immobile + TALL immobile) more than 50% of the time. The time fraction of Thy1 immobility was 29%, which is consistent with the immobile time fraction previously found in HeLa cells (~20%; [22]).

The time fractions of the mobile periods (and thus the immobile periods) for 1- and 2-wk neurons were not significantly different (for both PrP and Thy1), and therefore, the data for 1- and 2-wk neurons were combined (Fig. 2, bottom figure). Importantly, in more than 70% of the entire durations of the trajectories obtained here, PrP was immobile in 1 + 2-wk neurons. Even Thy1 exhibited a 52% immobile time fraction in neurons. However, in all cell types examined here, PrP always exhibited statistically significant larger immobile time fractions than Thy1 (by a factor of 1.9 in CHO-K1 cells and 1.4 in 1 + 2-wk neurons). Furthermore, both molecules exhibited higher immobile time fractions in 1 + 2-wk neurons, as compared with those in CHO-K1 cells. More detailed quantitative data are summarized in Table 1.

**Table 1:** The time fractions of the mobile and immobile (all-time immobile + TALL) periods of PrP and Thy1. The difference in the mobile fractions between CHO-K1 vs. 1 + 2-wk neurons was statistically significant, with *P* values of 0.0026 for PrP and 0.0037 for Thy1 (*P* values obtained by the Mann-Whitney *U*-test)

Cells/molecules	Time fraction ± SEM (%)		*P* value of PrP vs. Thy1 (mobile fraction)	Total examined period (s)	Number of cells (*n*)
	Immobile	Mobile			
CHO-K1 cells					
PrP	53.9 ± 3.6	46.1 ± 3.6	<0.001	364.9	23
Thy1	28.8 ± 4.7	71.2 ± 4.7		266.1	18
1-wk neurons					
PrP	71.1 ± 3.4	28.9 ± 3.4	﻿ ﻿﻿ 0.023	165.6	10
Thy1	48.5 ± 8.2	51.5 ± 8.2		54.0	5
2-wk neurons					
PrP	71.8 ± 5.6	28.2 ± 5.6	0.037	72.5	5
Thy1	54.8 ± 4.4	45.2 ± 4.4		112.3	5
1 + 2-wk neurons					
PrP	71.4 ± 2.8	28.6 ± 2.8	0.0017	238.1	15
Thy1	51.7 ± 4.5	48.3 ± 4.5		166.3	10

The occurrences of such high time fractions of immobile periods are quite unusual among various membrane-integrated molecules in the PM. Transmembrane proteins, such as transferrin receptor [35] and GPCRs [27, 41, 42] located outside coated pits, as well as phospholipids and gangliosides [13], exhibited transient immobile periods, but their time fractions are less than 5%.

### Effective Diffusion Coefficient of PrP Is Smaller than that of Thy1 by a Factor of 2.1–4.5

**Fig. 3:**
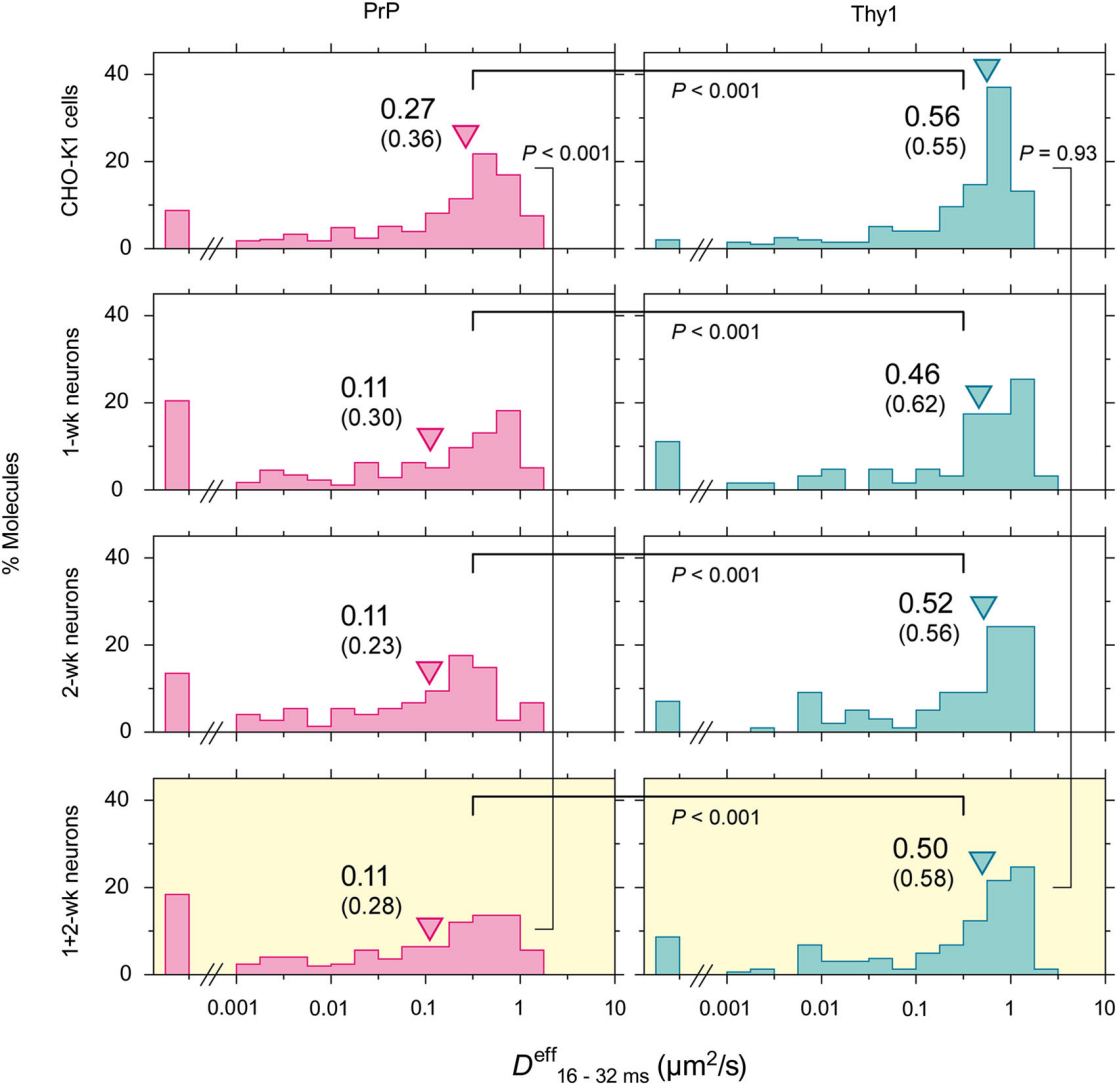
The distributions of the effective diffusion coefficients in the time scale between 16 and 32 ms (*D* ^eff^_16–32ms_) of single individual PrP and Thy1 trajectories (*left and right columns*, respectively). The *triangles* and the *numbers* next to them represent median values (in µm^2^/s). The *numbers in parentheses* indicate mean values. *P* values represent the results of the Mann-Whitney *U*-test. Further details of the *D* ^eff^_16–32ms_ data are shown in Table 2

The distributions of the diffusion coefficients of single individual PrP and Thy1 trajectories, obtained for the time scale between 16 and 32 ms (*D* ^eff^_16–32ms_), are shown in Fig. 3. As described in the Materials and Methods, since the diffusion coefficient for each molecule was calculated by using the distances of all possible pairs of the molecules’ coordinates in each trajectory between (*x*_i_, *y*_i_) and (*x*_i+*n*_, *y*_i+*n*_), where i indicates the ith point in the trajectory and *n* = 2, 3, and 4 (molecular step sizes for the time intervals of 16, 24, and 32 ms), this diffusion coefficient for each molecule represents an average diffusion coefficient over the entire trajectory, including both the mobile and immobile periods. Therefore, such an average diffusion coefficient is useful to estimate the rate for long-term, long-range diffusion (4 × *D* ^eff^_16–32ms_ × [time interval] describes the size of the 2D area covered by the molecule during a given time interval), and thus is termed the “effective” diffusion coefficient (*D* ^eff^_16–32ms_).

In all of the cell types employed here (CHO-K1 and 1- and 2-wk neurons), the *D* ^eff^_16–32ms_ of PrP is significantly smaller than that of Thy1. The differences were approximately a factor of 2.1 in CHO-K1cells and a factor of 4.5 in 1- and 2-wk neurons. No significant difference was found between 1- and 2-wk neurons, and thus their results were combined (Fig. 3, bottom boxes with yellow highlighting). PrP in 1 + 2-wk neurons exhibited significantly smaller *D* ^eff^_16–32ms_, as compared with that in CHO-K1 cells, by a factor of 2.5 (comparison of median values). Meanwhile, the diffusion of Thy1 did not show any sign of variations for the different cell types. These results suggest the abnormal homo-protein and hetero-protein interactions of PrP in neuronal cells. Further details of the data are provided in Table 2.

**Table 2:** The effective diffusion coefficients in the time scale between 16 and 32 ms (*D* ^eff^_16–32ms_) of single individual PrP and Thy1 trajectories. The difference between 1-wk vs. 2-wk neurons was statistically insignificant for both PrP and Thy1 (using the Mann-Whitney *U*-test), and therefore, the results are combined at the bottom of this table. The difference between CHO-K1 vs. 1 + 2-wk neurons in *D* ^eff^_16–32ms_ was statistically significant for PrP (*P* < 0.001), but not for Thy1 (*P* = 0.93). *P* values in the table are for comparing PrP and Thy1 in the same cell type, and show the results of the Mann-Whitney *U*-test

Cells/molecules	Median (μm^2^/s)	Mean ± SEM (μm^2^/s)	*P* of PrP vs. Thy1	Number of events (*n*)
CHO-K1 cells				
PrP	0.27	0.36 ± 0.020	<0.001	331
Thy1	0.56	0.55 ± 0.029		197
1-wk neurons				
PrP	0.11	0.30 ± 0.028	<0.001	176
Thy1	0.46	0.62 ± 0.073		63
2-wk neurons				
PrP	0.11	0.23 ± 0.037	<0.001	74
Thy1	0.52	0.56 ± 0.048		99
1 + 2-wk neurons				
PrP	0.11	0.28 ± 0.023	<0.001	250
Thy1	0.50	0.58 ± 0.041		162

### Characteristics of Individual Immobilization Events

Next, we examined the durations of the individual immobile events (observed for each single molecule). The raw data (*τ*_obs_) are summarized in Table 3a. Comparing PrP and Thy1 in the same cell type, the immobile durations were quite similar to each other: ~0.7 s in CHO-K1 cells and 0.8~1 s in 1- and 2-wk neurons (the durations exhibited by PrP and Thy1 were statistically significantly different only in 2-wk neurons, but the difference was small). The differences in the immobile and mobile durations of PrP and Thy1 among the three cell types were complex, and will not be discussed in this report.

**Table 3:** The durations of the individual immobile (**a**) and mobile (**b**) events (observed for each single molecule). *P* values are for comparing PrP and Thy1 (in terms of the duration of each period of the immobile and mobile states) in the same cell type, and show the results of the Mann-Whitney *U*-test. The differences in the immobile and mobile durations of PrP and Thy1 among the three cell types were complex, and will not be discussed in this report

a. The durations of immobile states				
Cells/molecules	Duration of each immobilaiton event (*τ*_obs_) (Mean ± SEM [s])	*P* for PrP vs. Thy1	Duration of each immobilation event after correction for photobleaching (*τ*_immob_) (Mean ± SEM [s])	Number of events (*n*)
CHO-K1 cells				
PrP	0.67 ± 0.053	0.98	1.74 ± 0.36	289
Thy1	0.69 ± 0.090		1.84 ± 0.65	131
1-wk neurons				
PrP	0.89 ± 0.076	0.56	4.69 ± 2.20	133
Thy1	0.87 ± 0.19		4.11 ± 4.29	31
2-wk neurons				
PrP	0.79 ± 0.12	0.018	2.75 ± 1.43	67
Thy1	0.99 ± 0.14		10.41 ± 15.02	62

b. The durations of mobile states				
Cells/molecules	Duration of each mobile event (Mean ± SEM [s])	*P* for PrP vs. Thy1	Duration of each mobile event after correction for photobleaching (Mean ± SEM [s])	Number of events (n)
CHO-K1 cells				
PrP	0.45 ± 0.032	<0.001	0.75 ± 0.091	381
Thy1	0.70 ± 0.053		1.91 ± 0.41	252
1-wk neurons				
PrP	0.30 ± 0.026	0.0064	0.41 ± 0.049	158
Thy1	0.46 ± 0.070		0.79 ± 0.21	59
2-wk neurons				
PrP	0.28 ± 0.041	<0.001	0.38 ± 0.074	70
Thy1	0.52 ± 0.052		0.99 ± 0.19	97

The raw lifetime (*τ*_obs_) obtained from the experiments listed in Table 3a represents both the rate at which the molecule transits from immobile to mobile states and the rate of photobleaching, in the relationship of 1/*τ*_obs_ = 1/*τ*_immob_ + 1/*τ*_photobleaching_, where *τ*_immob_ and *τ*_photobleaching_ represent the (correct) duration of the immobile period and the photobleaching lifetime, respectively. Using the notation of *k*_M_ and *k*_photobleaching_ described in the text to explain the three trajectory categories, 1/*τ*_immob_ = *k*_M_ and 1/*τ*_photobleaching_ = *k*_photobleaching_ (*k*_M_ represents the kinetic constant for the transition from the immobile state to the mobile state, and *k*_photobleaching_ represents the kinetic constant for photobleaching). Therefore, 1/*τ*_obs_ can be written as *k*_M_ + *k*_photobleaching_. Note that even when we measured the duration of all-time immobile molecules from time 0 until the time of photobleaching, it includes the rate of the transition from the immobile state to the mobile state.

Under our observation conditions, *τ*_photobleaching_ was 1.10 ± 0.037 s or 138 frames for ATTO594 employed in this study. Therefore, the correct immobile lifetime (*τ*_immob_) can be obtained from the raw lifetime, using the equation 1/*τ*_immob_ = 1/*τ*_obs_−1/*τ*_photobleaching_ or *τ*_immob_ = 1.10 × *τ*_obs_/(1.10−*τ*_obs_). *τ*_immob_ values are summarized in Table 3a.

Comparing PrP and Thy1 in the same cell type, the immobile durations were quite similar to each other in CHO-K1 cells and 1-wk neurons, with a mean *τ*_immob_ ~1.8 s in CHO-K1 cells and a mean *τ*_immob_~4.4 s in 1-wk neurons (mean of PrP and Thy1). By some unknown reasons, the Thy1 data obtained in 2-wk neurons included large variations and/or errors (10.41 ± 15.02 s). This is probably because a *τ*_obs_ value (0.99 s) was quite close to the photobleaching lifetime (1.10 s). Therefore, we cannot make any conclusions from the Thy1 data obtained in 2-wk neurons (our Thy1 data contained occurrences of several very long anchorage events, which increased the mean *τ*_obs_. Such very long anchorage events might be induced by large variations in the developmental stages of each cell at around 14 DIV, and/or by Thy1 interaction with astrocyte α_v_β_3_ integrin, which might start occurring at around 14 DIV [43]. Therefore, here, we decided to simply report what we observed, without interpretation). Meanwhile, a *τ*_immob_ value of 2.8 (±1.4) s was obtained for PrP in 2-wk neurons, which is quite comparable to a *τ*_immob_ value of 4.7 (±2.2) s for PrP in 1-wk neurons.

The sizes of the immobile sites were evaluated. During the immobile periods, the molecules still exhibited a jittering motion (true motion + apparent motion due to limited single-molecule localization precisions, but without macroscopic diffusion). The coordinates of these positions during an immobile period were fitted by a 2-dimensional Gaussian function, and the standard deviation was adopted as the radius of the immobile sites (Table 4). The radii were 50 nm in CHO-K1 cells and 39 nm in neurons. To obtain these values, all of the results obtained for PrP and Thy1 in CHO-K1 cells and those obtained in 1 + 2-wk neurons were combined, because no statistically significant differences were detected between PrP and Thy1 in any cell types and between 1- and 2-wk neurons.

**Table 4:** The size (Gaussian radius) of the sites for immobilization. Note that no statistical difference was detected between PrP and Thy1 in all three cell types. Since no statistical difference was detected between 1- and 2-wk neurons, the overall average of all TALL events in neurons is shown at the bottom of this table. The differences in the sizes of the immobilization sites between CHO-K1 cells and 1 + 2-wk neurons was significant (*P* < 0.001). The sizes shown in this table include a single-molecule localization precision of 37.3 ± 0.49 nm in 2D (see the main text). The actual sizes of the anchorage sites can be obtained by subtracting this precision value. They are 12.1 ± 1.0 and 1.7 ± 1.2 nm in radius (24.2 ± 2.0 and 3.5 ± 2.4 nm in diameter) in CHO-K1 cells and neurons, respectively

Cells/molecules	Gaussian radius; detailed results (Mean ± SEM [nm])	Number of immobile and TALL events (*n*)	Gaussian radius; final results (Mean ± SEM [nm])
CHO-K1 cells			
PrP	48.6 ± 1.1	289	49.4 ± 0.89
Thy1	51.2 ± 1.5	131	
1-wk neurons			
PrP	37.9 ± 1.6	133	37.4 ± 1.5
Thy1	35.2 ± 3.1	31	
2-wk neurons			
PrP	41.9 ± 2.4	67	41.0 ± 1.6
Thy1	40.0 ± 2.2	62	
1 + 2-wk neurons			
PrP	39.3 ± 1.4	200	39.0 ± 1.1
Thy1	38.4 ± 1.8	93	

Meanwhile, the localization precision of single ATTO594 molecules bound to the cover glass was 37.3 ± 0.49 nm in radius (SD of the 2D Gaussian function; 24.6 ± 0.47 and 28.0 ± 0.51 nm in horizontal and vertical directions of the camera, respectively). Therefore, the actual anchorage domain size can be obtained by subtracting the localization precision from the measured radius of immobilization site, providing 24.2 ± 2.0 and 3.5 ± 2.4 nm in diameter in CHO-K1 cells and neurons, respectively (Table 4).

These results clearly indicated that the differences found in the mobile vs. immobile time fractions and the effective diffusion coefficient *D* ^eff^_16–32ms_ between PrP and Thy1 and between CHO-K1 and neurons were not due to the properties of each immobilization event (with certain reservations due to the Thy1’s *τ*_immob_ results in 2-wk neurons). Therefore, next we examined the diffusion coefficient during mobile periods and the frequency of TALL occurrences (duration of each mobile period).

### Diffusion Coefficient during Mobile Periods of PrP Is Smaller than that of Thy1 by Factors of 1.5–1.7

**Fig. 4:**
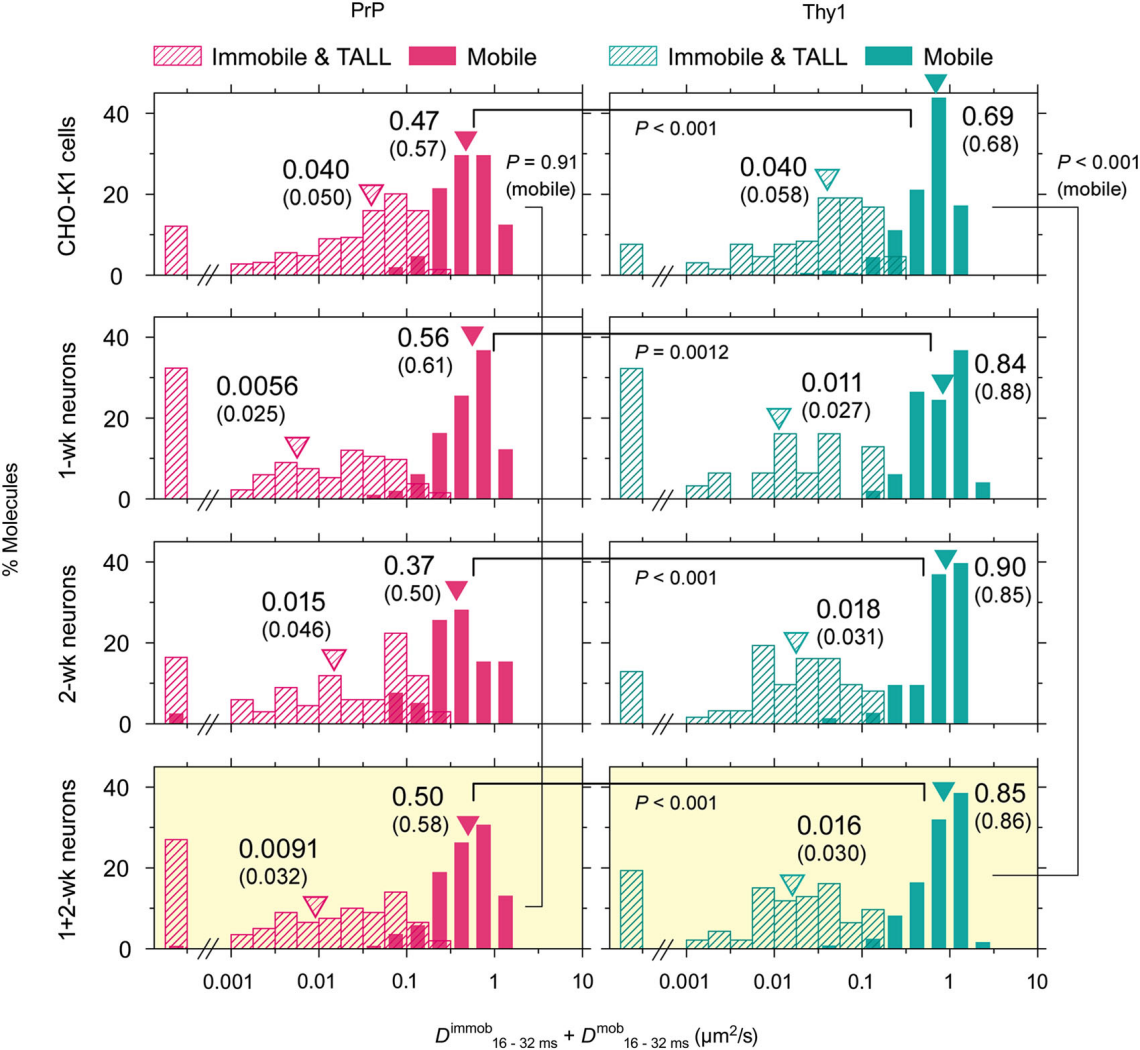
The distributions of the diffusion coefficients in the time scale between 16 and 32 ms during the mobile periods and immobile periods (*D* ^mob^_16–32ms_ and *D* ^immob^_16–32ms_, respectively) of single individual PrP and Thy1 molecules (*left and right columns*, respectively). *P* values represent the results of the Mann-Whitney *U*-test. The distributions of *D* ^immob^_16–32ms_ are shown only for reference purposes, because the results are likely dominated by variations of the noise level for individual fluorescent spots in each image frame (single-molecule localization precisions). Further details of the *D* ^mob^_16–32ms_ data are shown in Table 5

The distributions of the diffusion coefficients in the time scale between 16 and 32 ms during the mobile periods (*D* ^mob^_16–32ms_) are shown in Fig. 4. Interestingly, even during the mobile periods, *D* ^mob^_16–32ms_ of PrP is smaller than that of Thy1 by a factor of 1.5 in CHO-K1 cells and 1.7 in 1 + 2-wk neurons (since no statistically significant difference in *D* ^mob^_16–32ms_ was found for 1- and 2-wk neurons, their results were combined). The reason for this difference between PrP and Thy1 is not clear. We suspect that ACP-PrP has a higher propensity to form transient homodimers with non-labeled endogenous PrP, as compared to the propensity of ACP-Thy1 to form transient homodimers with non-labeled endogenous Thy1. In fact, Suzuki et al. [11] previously found that all of the GPI-ARs they examined, including Thy1, CD59, DAF, and GFP-GPI, frequently form transient homodimers with lifetimes on the order of 0.2 s, which work as units for raft organization and function.

If such transient homodimers occur in the PM, then the diffusion coefficient would be reduced, due to “oligomerization-induced trapping” [44]; i.e., the residency times of dimers and oligomers within the PM compartments (induced by the actin-based membrane skeleton and the transmembrane proteins bound to it) would be prolonged relative to those of monomers, because the hop probabilities of dimers and oligomers are reduced [39].

Furthermore, using immunoelectron microscopy, Brügger et al. [18] showed that PrP forms homo-clusters that occur quite independently from another GPI-AR, Thy1. If the fluorescent ACP-PrP we observed here became associated with PrP clusters, then it might become immobilized if the PrP cluster is immobile or exhibit slow diffusion if the PrP cluster is undergoing slow diffusion.

The cell dependence was quite curious. The *D* ^mob^_16–32ms_ values of PrP were the same in both CHO-K1 and 1 + 2-wk neurons. In contrast, the *D* ^mob^_16–32ms_ values of Thy1 were greater in 1 + 2-wk neurons than in CHO-K1 cells, by 23%. Further detailed data are provided in Table 5.

**Table 5:** The diffusion coefficients in the time scale between 16 and 32 ms during the mobile periods (*D* ^mob^_16–32ms_) of single individual PrP and Thy1 trajectories. The difference between 1-wk vs. 2-wk neurons was statistically insignificant for both PrP and Thy1 (using the Mann-Whitney *U*-test), and therefore, the results are combined at the bottom of this table. The difference in *D* ^eff^_16–32ms_ between CHO-K1 vs. 1 + 2-wk neurons was statistically significant for Thy1 (*P* < 0.001), but not for PrP (*P* = 0.91). *P* values in the table are for comparing PrP and Thy1 in the same cell type

Cells/molecules	*D* ^mob^_16–32ms_ median (μm^2^/s)	*D* ^mob^_16–32ms_ mean ± SEM (μm^2^/s)	*P* of PrP vs. Thy1	Number of events (*n*)
CHO-K1 cells				
PrP	0.47	0.57 ± 0.021	<0.001	256
Thy1	0.69	0.68 ± 0.026		180
1-wk neurons				
PrP	0.56	0.61 ± 0.037	0.0012	98
Thy1	0.84	0.88 ± 0.069		49
2-wk neurons				
PrP	0.37	0.50 ± 0.063	<0.001	39
Thy1	0.90	0.85 ± 0.045		73
1 + 2-wk neurons				
PrP	0.50	0.58 ± 0.032	<0.001	137
Thy1	0.85	0.86 ± 0.038		122

The distributions of the diffusion coefficients of PrP and Thy1 during immobile periods are shown in Fig. 4. However, since the diffusion coefficients calculated for immobile periods (*D* ^immob^_16–32ms_’s) are dominated by the noise (single-molecule localization precision) and jittering motion, they are simply presented for the purpose of ascertaining that the immobile periods determined by the TALL detection method used here (essentially by the method developed by Sahl et al. [3]) were correct. Indeed, the *D* ^immob^_16–32ms_ values were much smaller than the diffusion coefficients during the mobile periods (*D* ^mob^_16–32ms_).

The *D* ^eff^_16–32ms_ value (effective diffusion coefficient averaged over both mobile and immobile periods) of Thy1 was greater than that of PrP, by a factor of 2.1. This could be mostly explained by the 1.5× longer mobile time fraction coupled with the 1.5× greater *D* ^mob^_16–32ms_. Likewise, the 4.5× greater *D* ^eff^_16–32ms_ of Thy1, as compared with that of PrP, in 1 + 2-wk neurons could be explained by the 1.7× longer mobile time fraction coupled with the 1.7× greater *D* ^mob^_16–32ms_ reasonably well.

### Individual Mobile Periods of PrP are Shorter than Those of Thy1 by a Factor of 1.9–2.6, Indicating More Frequent Occurrences of TALL Events

Next, we examined the durations of the individual mobile periods. The results are summarized in Table 3b. Importantly, the average duration for each mobile period of PrP was shorter than that of Thy1 by a factor of 1.9~2.6 (values after the correction for photobleaching). Considering the result that the average durations for each immobile period were the same for both PrP and Thy1, this result for each mobile period indicates that the greater time fraction of the immobile period of PrP, as compared to that of Thy1, is primarily due to the shorter individual mobile periods (rather than longer durations for individual immobile periods); i.e., the more frequent occurrences of TALL events of PrP.

## Discussion

Both PrP and Thy1 exhibited intermittent transient immobilization events. Each immobilization event lasted for a few seconds, with an immobilized area size of 24.2 and 3.5 nm in CHO-K1 and neurons, respectively. In 1 + 2-wk neurons, PrP molecules were immobile for 72% of the time (which is the same as 72% of the molecules were immobile at any given moment), whereas the immobile time fraction for Thy1 was 52%. These time fractions were 54 and 29% for PrP and Thy1, respectively, in CHO-K1 cells.

The occurrences of such high time fractions of immobilized periods and short periods of individual immobilization events were both quite unexpected in the PM. Brügger et al. [18] reported immunoelectron microscopy results suggesting that each PrP cluster might consist of at least several PrP molecules, and that many PrP clusters exist in the PM. Furthermore, they reported that the domains containing Thy1 clusters also tend to contain PrP, whereas the domains of PrP clusters essentially lack Thy1 molecules. Even when PrP clusters contain Thy1, it is only associated with their peripheral regions, suggesting the stronger molecular interaction of PrP as well as the enhanced interaction of PrP clusters. Taken together with the present results, PrP molecules tend to transiently interact with other PrP molecules, undergoing slower diffusion than Thy1, and with PrP clusters, perhaps becoming transiently entrapped in or associated with PrP clusters, and thus undergoing temporary cessation of lateral diffusion. The PrP clusters themselves would be dynamic, and their constituent PrP molecules might be newly recruited to and associated with PrP clusters all the time, and simultaneously, the constituent PrP molecules might be continuously departing from the PrP clusters (Rouvinski et al. [45] demonstrated the misfolded PrP was present in the PM in the form of strings or webs that remain stable for long periods. Our data cannot address the result described by Rouvinski et al., but our results suggest a possibility that the constituent misfolded PrP molecules in string or web structures might be undergoing rapid exchanges with those in the bulk PM). Thy1 would have similar tendencies, but its homo-interactions would be weaker than those between PrP molecules.

Meanwhile, it should be noted that the residency lifetime of PrP molecules in PrP clusters and the size of PrP clusters (possibly domains made of PrP clusters) are not very different from those of Thy1. However, the frequency of PrP becoming entrapped in (associated with) the PrP clusters (domains) is substantially higher than that of Thy1 existing in (or with) the Thy1 clusters (and the domains formed by Thy1 clusters), suggesting higher on-rates (faster association kinetics) of PrP–PrP binding than Thy1–Thy1 binding. Consistently with these observations, preliminary experiments of homo-crosslinking PrP or Thy1 by the addition of primary antibodies (Halo-PrP and Halo-Thy1 were fluorescently-labeled with TMR and they were crosslinked by anti Halo antibodies; Halo-tagged proteins exhibited virtually the same diffusion properties as ACP-tagged proteins) showed that anti Halo antibody-induced crosslinking increased the immobile time fraction of Thy1 more extensively than that of PrP. This result suggests that PrP is already quite well immobilized by PrP homo-interactions under control conditions, whereas Thy1, which exhibited more mobile fractions under control conditions, could be induced to become more immobile.

The propensities of PrP to form transient oligomers and clusters might be the basis for the rapid transmission of pathogenic misfolding, from misfolded PrP to normal PrP. Goold et al. [16] previously demonstrated that prion infection of cells is rapid occurring within 1 min of prion exposure, and that the PM is the primary site of prion conversion. How can such fast spread of infection on the cell surface be induced? The present results indicate that, if misfolded PrP could associate with PrP oligomers, it would lengthen the interaction time between misfolded PrP and normal PrP by a factor of a million or more, due to the prolongation of the interaction durations perhaps from less than microseconds to a few seconds, greatly increasing the opportunity for infection to be established. The dynamic association (the short lifetime of the PrP clusters) would allow the movement of PrP from cluster to cluster, further enhancing propagation of the infection on the cell surface. Therefore, we propose that, due to the ability and fast kinetics of dimer and oligomer formation by normal PrP, the interaction of misfolded PrP with normal PrP might be greatly enhanced, inducing the rapid propagation of PrP misfolding in the PM. Although we are currently not equipped to perform experiments using misfolded PrP to test this proposal, we set the basis for future studies examining this hypothesis.

## Abbreviations

ACP: Acyl-carrier protein
DIV: Days in vitro
GPI-AR: Glycosylphosphatidylinositol-anchored receptor
PrP: Prion protein
PM: Plasma membrane
STALL: Stimulation-induced temporary arrest of lateral diffusion
TALL: Temporary arrest of lateral diffusion
